# Mechanical Ventilation, Retinal Avascularity and Rate of Vascularisation: A Triad of Predictors for Retinopathy of Prematurity Treatment

**DOI:** 10.3390/jpm14040379

**Published:** 2024-03-31

**Authors:** Olena Protsyk, José Luis García Serrano

**Affiliations:** 1 Department of Ophthalmology, Jaen University Hospital, Av. del Ejército Español 10, 23007 Jaén, Spain; olenaprotsyk@correo.ugr.es; 2 Department of Surgery and Related Specialities, University of Granada, 18012 Granada, Spain; 3 Ophthalmology Service, Hospital Clínico San Cecilio, 18016 Granada, Spain

**Keywords:** retinopathy of prematurity, treatment, predictive model, avascular area, mechanical ventilation, risk tables, rate of vascularisation

## Abstract

Aim: The temporal avascular area of the retina and the duration of mechanical ventilation (DMV) may predict the need to treat retinopathy of prematurity (ROP). This study considers whether the rate of retinal vascularisation and related risk factors should be included in a predictive model of the need for ROP treatment. Methods: This single-centre, observational retrospective case–control study was conducted on 276 preterm infants included in an ROP screening programme. All had undergone at least three examinations of the fundus. The main outcome measures considered were DMV (in days of treatment), the temporal avascular area (in disc diameters, DD) and the rate of temporal retinal vascularisation (DD/week). Results: The multivariate logistic model that best explains ROP treatment (*R*^2^ = 63.1%) has three significant risk factors: each additional day of mechanical ventilation (OR, 1.05 [95% CI, 1.02–1.09]; *p* = 0.001); each additional DD of temporal avascular area (OR, 2.2 [95% CI, 1.7–2.9]; *p* < 0.001) and a vascularisation rate <0.5 DD/week (OR, 19.0 [95% CI, 6.5–55.5]; *p* < 0.001). Two tables are presented for calculating the expected need for ROP treatment according to these three risk factors. Conclusions: A greater DMV, a broad avascular area of the temporal retina at the first binocular screening and slow retinal vascularisation strongly predict the need for ROP treatment. The predictive model we describe must be validated externally in other centres.

## 1. Introduction

Retinopathy of prematurity (ROP) is considered a disease of retinal vascular proliferation affecting preterm undergoing oxygen therapy. During phase 1 of ROP, delayed developmental retinal vascularisation occurs, and some eyes have a loss of retinal capillary network. Damaged newly developed vessels appear in the vascular–avascular area. In phase 2 ROP, increased VEGF due to retinal ischemia facilities vascular growth. Most preterm achieve complete vascularisation of the retina. In the advanced stage of ROP, wide vascular areas are the trigger that favours an overproduction of growth factors, particularly VEGF and the development of extraretinal neovascularization. Therefore, we should prevent ROP progression mainly focussing on avoiding retinal detachment with early ROP treatment.

Among the many risk factors associated with the need for ROP treatment [1] are low gestational age, low birth weight, poor weight gain, postnatal sepsis, transfusions, multiple births, bronchopulmonary dysplasia and perinatal morbidity [2]. Other factors that have been studied in this context include fluctuations in oxygenation, high levels of oxygen concentration and prolonged mechanical ventilation [3]. Some predictive models include the latter variable [4]. Weight gain has been used in various algorithms, presenting high sensitivity, to predict severe ROP, thus reducing the number of examinations needed [5].

Abnormal vascularisation in ROP may be associated with risk factors located in the retina such as a broad avascular area and slow vascularisation [6,7,8]. Among other factors, the area of incomplete vascularisation is determined by extreme prematurity and by other conditions that may inhibit normal retinal vascular growth such as hyperoxia [9,10]. The greater the area of incomplete vascularisation (zone I-II), the greater the risk of retinal neovascularisation [11]. At >32 weeks of gestational age, stage II or proliferative ROP begins [12]. The longer the duration of ischaemia within this phase, the greater the risk of severe ROP [13]. A low rate of vascular growth (i.e., the speed of temporal retina vascularisation), defined as <0.54 DD/w by Pahdi [7] or −0.5 DD/w by Solans Pérez de Larraya [8], also increases the risk that ROP treatment will be needed. When ROP does not require treatment, the greater the vascular development, the more likely and the faster the natural involution of the disease [14].

The aim of the present study is to determine the association between ROP requiring treatment and its risk factors and to perform a predictive model of this condition. The study variables include those directly related to the development of retinal vasculature, such as the avascular area, the rate of vascularisation and the rate of vascular outgrowth.

## 2. Materials and Methods

### 2.1. Study Population

**Figure 1:**
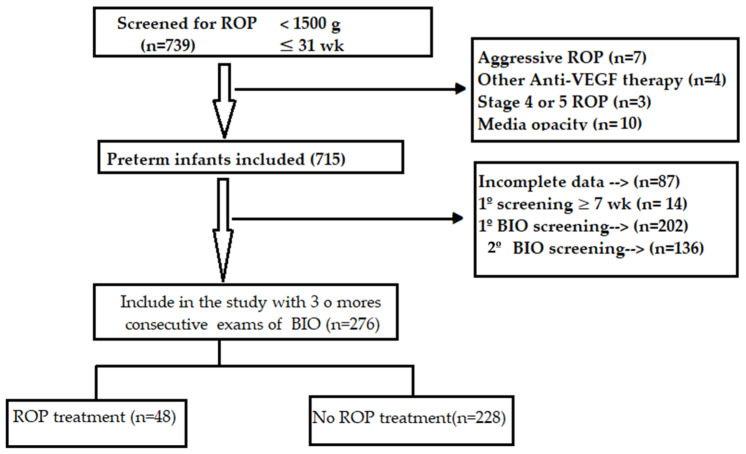
Participant flow diagram. BIO, binocular indirect ophthalmoscopy. 1º BIO screening, at the first examination full vascularization of the retina or ≤1 DD temporal avascular area. 2º BIO screening, full vascularization or ≤1 DD.

A retrospective case–control study was performed with premature infants born at the San Cecilio University Hospital in Granada (Granada, Spain) during the period 2002–2022. Structured data were collected for all infants screened for retinopathy of prematurity (ROP) (*n* = 739) under the following criteria: birth weight < 1500 g or gestational age (GA) ≤ 31 weeks, birth weight between 1501 and 2000 g and GA ≥ 32 weeks with oxygen supply > 72 h, or unstable clinical course as determined by the neonatologist [15] (Figure 1).

This study is focused on premature newborns who underwent at least three fundus examinations (to ensure sufficient data with which to calculate the rate of vascularisation) with full follow-up until discharge. The following exclusion criteria were applied: failure to examine preterm infants during weeks 4–6 postnatal, media opacity and stage 4 or 5 ROP or aggressive ROP. In addition, four infants treated with anti-vascular endothelial growth factor were excluded because of an insufficient sample size. In total, 276 premature infants were included in the study, which was approved by the Biomedical Research Ethics Committee of Andalusia in February 2021 (Reg. no. 0028-N-21). Written informed consent was obtained from the parents or guardians of all infants included in the study.

Of the total of 48 patients treated for ROP from 2002 to 2005, most of their eyes met the threshold ROP criteria (*n* = 6): 5 contiguous or 8 cumulative clock hours of stage 3 ROP in zone I or zone II, with plus disease. From 2005 to 2022, the treatment criterion was ETROP Type 1 Prethreshold ROP in zone I and II, 2+ 3+ (*n* = 40). Two patients have been treated in zone III, 3 with persistent avascular retina.

### 2.2. Ophthalmologic Examination

**Figure 2:**
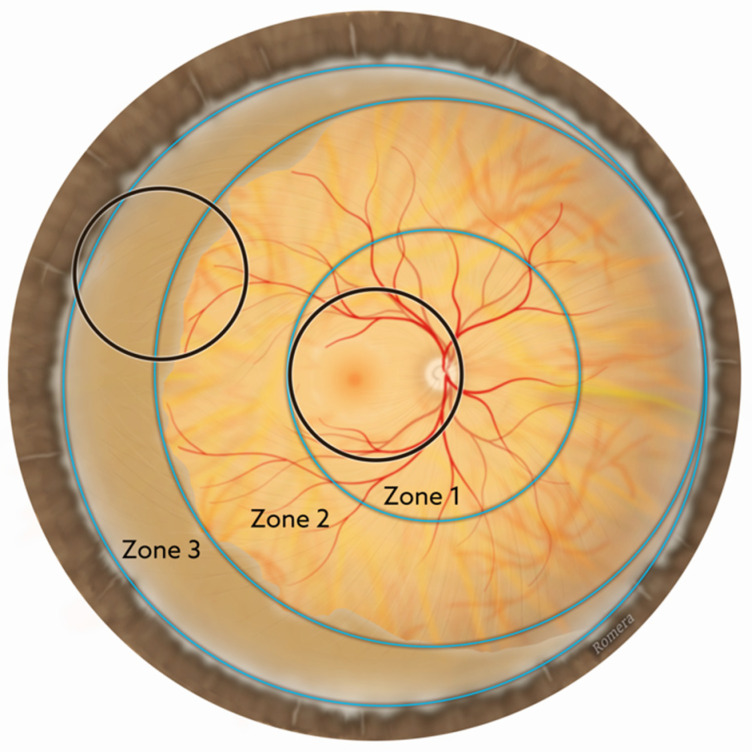
Zone 3 of the peripheral retina is avascular. With binocular ophthalmoscopy, the black circles represent the field of view with a 28 dioptre lens.

All patients were examined by the same paediatric ophthalmologist, using binocular indirect ophthalmoscopy (BIO) with indentation (Figure 2), after administering dilating agents (phenylephrine 1% and cyclopentolate 0.2%) and anaesthetic drops. The BIO device used was fitted with a video system to capture the images obtained.

The first screening examination for ROP was performed at postnatal week 4. Scans were performed weekly or every 2 weeks [15,16,17].

All clinical data were recorded from inclusion of the preterm infants in the screening protocol for ROP until medical discharge. After each fundus examination, ophthalmological notes were taken detailing the severity of ROP (stage), the affected zone (I–III), the extent in clock hours, the presence of preplus or plus disease and the extent of avascular retina in disc diameter [17,18]. A random sequence was performed for the selection of the studied eye (right or left).

**Figure 3:**
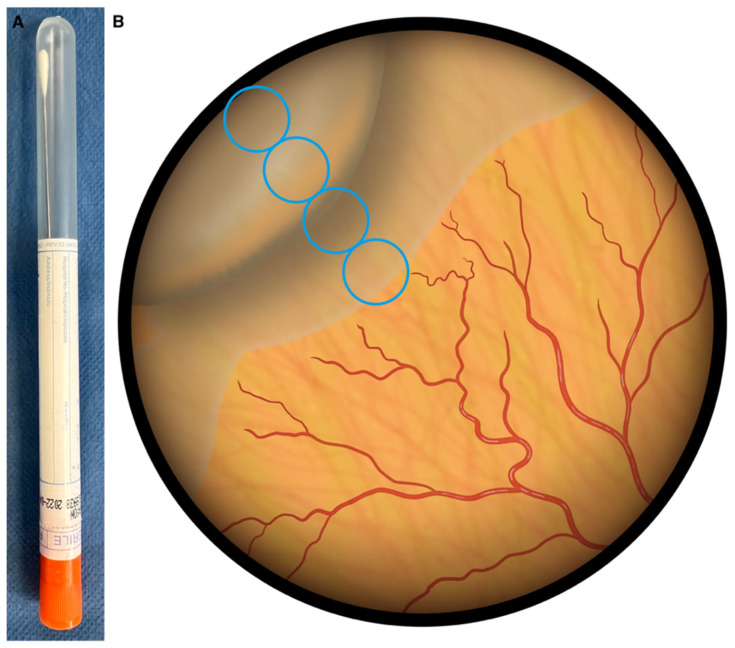
(**A**) Sterile aluminium swab with cotton used for indentation. (**B**) Technique for quantifying the peripheral avascular retina with binocular indirect ophthalmoscopy and a 28 dioptre lens. The unit of measurement is the horizontal DD. The indentation of the swab is equal to 2 DD at the top left. The diameter of the circle of the visible area with a 28 dioptre lens is equivalent to 8 DD. The distance from the temporal avascular area to the periphery of the retina is 3.5 DD.

The avascular area extends from the retinal vascular development border to the periphery. All explorations of the avascular area were performed in a centripetal direction. The width was measured from the ora serrata to the temporal retina, approximately on the imaginary horizontal line passing through the centre of the papilla and the fovea, and taking the horizontal diameter of the disc (DD) as the unit of measurement. The lens used was the Volk 28 diopter BIO lens, which provides a field of view of approximately 55°, equivalent to about 8 DD. This proportion was checked at each examination. To quantify the more peripheral avascular retina, the degree of indentation of the eyeball was determined, using a dry swab with an aluminium applicator (wrapped swabs 160C, COPAN^®^, Murrieta, CA, USA) (Figure 3A). The first step in this process is to clearly visualise the grey prominence of the scleral depression (Figure 3B). At the lateral canthus, this is usually equivalent to 2 DD with the depressor used. From this reference, the avascular area can be quantified up to the demarcation line or until the first temporal vessel is visualised. Sometimes, to visualise a vessel, it is helpful to move the scleral depressor slightly parallel to the limbus. In the 4th postnatal week, the temporal avascular area of the retina was measured in optic disc diameters (DD), and recorded with differences of 0.5 DD.

The vascularisation rate is determined after three or more assessments, while keeping the window of study time as wide as possible. It is calculated in two ways: (1) the distance from the temporal periphery of the retina to the avascular area in DD, divided by the time in weeks necessary for its vascularisation; (2) the difference between the distance of the avascular area in DD (from the first scan to the day it is treated with laser) divided by the time in weeks from the first scan to the laser treatment.

### 2.3. Risk Factors for Retinopathy of Prematurity

The following risk factors for ROP were considered: gestational age (weeks), birthweight (grams), duration of invasive mechanical ventilation (days), nasal continuous positive airway pressure (days), rate of retinal vascularisation (DD/week), patent ductus arteriosus, sepsis, maternal age, weight gain (grams/day), Apgar score [19], the avascular area of the temporal retina (DD), the time to complete retinal vascularisation (weeks), and the presence or otherwise of newborn respiratory distress syndrome, patent ductus arteriosus [20], neonatal intracranial haemorrhage and/or periventricular leukomalacia (confirmed through neuroimaging) [21,22].

Also taken into account were the type of pregnancy (singleton or twin pregnancy) and the presence or otherwise of bronchopulmonary dysplasia (BPD), as defined and classified by Higgins et al. by severity (stage I, II and III) [23]. Considering that neonatal apnoea can be defined by duration or clinical repercussions, we defined it as a state in which the clinical instability of the infant prevents the ophthalmologic examination from being performed for at least one week [24]. The presence of sepsis was defined as an acute change in the sequential organ failure assessment score ≥ 2 points and a positive blood culture for fungi or bacteria [25].

### 2.4. Statistical Analysis

All statistical analyses were performed using Statistical Package for Social Sciences (SPSS 28.0; IBM Corp., Armonk, NY, USA). Categorical variables were compared using Pearson’s χ^2^ test or Fisher’s exact test. Normality of the numerical variables was determined by the Shapiro–Wilk test. Continuous variables were analysed descriptively and are expressed as mean ± standard deviation and as the median (P25, P75). When there were two study groups, they were compared using Student’s *t*-test or its non-parametric equivalent, the Mann–Whitney U-test. When there were three or more groups, we used either one-way ANOVA followed by post hoc multiple comparisons, or its non-parametric equivalent, the Kruskal–Wallis test. After univariate analysis, all significant factors were included in the multivariate analysis. The association between ROP requiring treatment and general or retinal variables was analysed using logistic regression. Multivariate models were constructed with a maximum of five variables. All tests were two-tailed, and *p* < 0.05 was considered to represent statistical significance.

## 3. Results

### 3.1. Participant Characteristics

For this analysis, the original study population consisted of 739 preterm infants. Of these, 463 failed to meet the inclusion criteria and were excluded. Thus, 276 premature infants were finally included in the study. Among this population, 48 (17.4%) received ROP treatment, although the condition was detected in 119 (43.1%). The following ROP types were observed: stage 1 (38; 13.8%); stage 2 (23; 8.3%); stage 3 (57; 20.7%); stage 5B (1; 0.4%).

The mean age of the mothers was 31.83 ± 5.9 years. In total, 52 (18.8%) had preeclampsia or eclampsia. In 214 cases (77.5%), delivery was by caesarean section. A twin pregnancy was recorded in 102 cases (37%). In 20 (7.2%) of these twin pregnancies, one of the siblings died. The median Apgar 1 score was 6 and the mean Apgar 2 score was 8.

The study population had a mean gestational age of 28.69 ± 2 weeks and a mean birth weight of 1077.23 ± 277 g. The mean retinal avascular area in the first examination was 3.47 ± 1.67 papillary diameters, with a maximum value of 8 DD. The mean rate of retinal vascularisation was 0.59 ± 0.27 disk diameters per week (DD/w), with a range from 0.007 DD/w to 1.5 DD/w. Of the premature infants, 185 (67%) had a mean rate of retinal vascularisation ≥0.6 DD/w, while 91 (33%) had a lower rate. The average duration of mechanical ventilation was 9.40 ± 3.77 days, that of continuous positive airway pressure was 9.04 ± 3 days and that of nasal cannula, 12.26 ± 3.7 days. The mean postnatal weight gain in the first four weeks was 12.42 ± 6 g/day.

The following values were obtained for the incidence of morbidities among survivors: apnoea *n* = 39 (14.3%), sepsis *n* = 113 (40.9%), patent ductus arteriosus *n* = 60 (21.7%), one or more transfusions performed *n* = 157 (57%). The incidence of mild–moderate bronchopulmonary dysplasia (BPD) was *n* = 88 (31.9%) and that of severe BPD, *n* = 132 (47.8%) [23]. The overall incidence of intraventricular haemorrhage was *n* = 41 (14.9%). Of these, 14 (5.1%) were grade 1, 19 (6.9%) were grade 2, 5 (1.8%) were grade 3, and 3 (1.1%) were grade 4.

### 3.2. Univariate Analysis of Risk Factors for ROP Treatment

Univariate analysis revealed no significant association between ROP treatment and the following risk factors: maternal age, hypertensive disorders of pregnancy, maternal drug use, caesarean delivery, Apgar score at minutes 1 and 5, sex, duration of high-flow nasal cannula, and methylxanthine therapy.

Univariate analysis showed that greater gestational age was a protective factor (OR = 0.637, *p* = 0.000), as were higher birth weight (OR = 0.996, *p* = 0.000) and good postnatal weight gain (OR = 0.938, *p* = 0.034). Transfusion (OR = 2.1, *p* = 0.030) and weight gain < 7.50 g per day (OR = 4.1, *p* = 0.000) at four weeks were both significantly associated with the need for ROP treatment.

**Figure 4:**
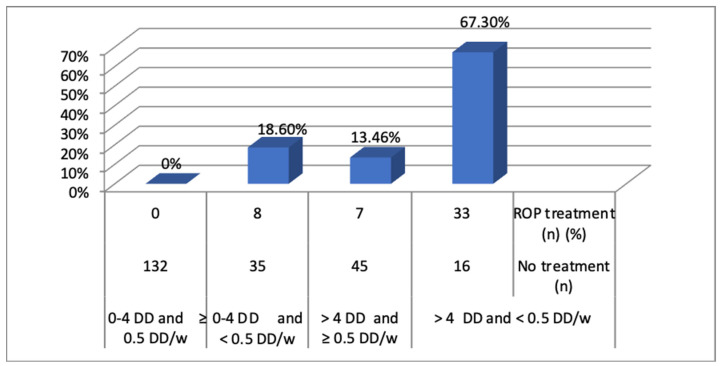
Association between the risk of ROP treatment vs. No treatment. w, week.

A greater area of the temporal avascular retina at four weeks (OR = 2.3, *p* = 0.000), especially ≥4 DD, is associated with a significantly higher risk of ROP treatment (OR = 14.0, *p* = 0.000). Slow retinal vascularisation, especially ≤0.5 DD/week, is also associated with a significantly higher risk of ROP treatment (OR = 20.8, *p* = 0.000) (Figure 4).

The risk increases with an avascular area >4 DD and with vascularisation <0.5 DD/week. The premature babies with an avascular area of 2–4 DD and good vascularisation (≥0.5 DD/week) did not require treatment for ROP (0%). However, treatment was required for 18.6% of those with ≤4 DD and <0.5 DD/week, for 13.5% of those with >4 DD and ≥0.5 DD/week, and for 67.3% of those with >4 DD and <0.5 DD/week.

The longer the duration of mechanical ventilation, the greater the probability of ROP treatment being needed (OR = 1.074 *p* = 0.000). Newborns who required mechanical ventilation for ≥12 days had a substantially increased risk in this respect (OR = 6.38, *p* = 0.000). The presence of apnoea also had a considerable impact on the likelihood of ROP treatment (OR = 10.5, *p* = 0.000).

### 3.3. Multivariate Analysis of ROP Treatment

The above-described statistical methods were used to generate the multivariate model (Table 1), which includes the duration of intubation oxygen therapy, the retinal avascular area recorded in the first examination, and the rate of vascularisation (as a qualitative variable) (R^2^ Nagelkerke: 63.1%, *p* = 0.000) (sensitivity: 60.4%, specificity: 95.2%). For selected cut-offs over 7–9 weeks postnatal age, this prediction model had an area under the receiver operating characteristic curve of 0.95 (95% CI, 0.92–0.97) and correctly classified cases with an accuracy of 89.10%.

Final equation of the logistic regression model:

Probability of ROP requiring treatment = 1/(1 + *e* − (−7.147 + 0.794(avascular area in DD) + 0.053(MV time in days) + 2.946 × 1(<0.5 DD/week))

In this equation, the rate of vascularisation <0.5 DD/week = 1, ≥0.5 DD/week = 0.

### 3.4. Tables for Calculating the Risk of ROP That Needs Treatment

Using the above model, we formulated risk tables corresponding to the two ranges of vascularisation considered (<0.5 DD/w vs. ≥0.5 DD/W). Both tables include invasive mechanical ventilation time and avascular area in DD as risk factors.

This table can be differentiated into three risk areas: With <4 DD of avascular area (very low risk of treated ROP), the good rate of vascularisation advances and occupies the ischaemic retinal area. With ≥7 DD, the good rate of vascularisation may be insufficient to compensate for the ischaemic retina and determine type 1 ROP, and the risk increases as the avascular area becomes more extensive and as more days of invasive mechanical ventilation are required. Finally, there is an intermediate zone with 5–6 DD of avascular area.

Table 2 shows that with a rate of vascularisation ≥0.5 DD, the risk of treated ROP appears when the avascular area is ≥4 DD. When the rate of vascularisation is <0.5 DD (Table 3), this risk is apparent at 2–3 DD.

## 4. Discussion

This study spans a period of 20 years, during which many protocols have changed (for example, in blood product transfusion procedures) and improvements have been made in our ICU, in areas such as nutrition and oxygen administration. The mean maternal age has risen slightly, and the percentage of premature babies born following in vitro fertilisation has increased. Moreover, in accordance with national and international guidelines, we have modified the protocols for monitoring and treating premature infants [15,16,17,18]. Despite these changes, the average gestational age of the children included in the ROP protocol remains above 29 weeks, the number of premature babies treated is stable at 35–40 per year, and these infants continue to be diagnosed, monitored and treated by the same paediatric ophthalmologist, at his referral hospital, using the same indentation system, based on binocular ophthalmoscopy and a 28 D lens.

The present study excludes premature infants treated with anti-VEGF therapy and those who present subsequent aggressive posterior ROP, as the number of such cases is insufficient for meaningful conclusions to be drawn. Nevertheless, the patients excluded from the analysis and those represented in the study present two factors in common. In both cases, there is a greater temporal avascular area of the retina and the disease is located in a more posterior position [28]. Furthermore, those who experienced arrested or delayed retinal vascular development often needed ROP treatment [29].

The ROP areas were classified using a binocular ophthalmoscope (BIO) and a 28 D lens, as the gold standard. Indeed, zone III is the peripheral retina usually visualised with the BIO and is almost never classified by image-based interpretation [30]. The BIO can also facilitate measurement of the retinal temporal avascular area (DD), which is quantified at increments of 0.5 DD (Figure 1 and Figure 2).

Gestational age (GA) and birth weight (BW) are major risk factors for ROP and are commonly included in screening protocols [31]. Predictive models based on these criteria usually seek to obtain a sensitivity of 100%. In our study, the inclusion criteria were BW ≤ 1500 g and/or GA ≤ 31 weeks. However, these criteria were insufficient because five infants, with GA = 31 weeks, required treatment but would not have been included in the analysis. Therefore additional criteria were applied to ensure that these patients were addressed by the screening protocol, which thus became infants with GA ≥ 32 weeks or BW < 2000 g and whom the attending neonatologist considered at risk of ROP [16,17,18].

Other predictive models of ROP are also based on GA, BW and lower than expected weight gain. In some cases, too, alarm systems are created to detect and/or refrain from excluding infants who require ROP treatment [32]. For example, the WINROP [33], CHOP-ROP [34] and CO ROP [35] algorithms for detecting type 1 ROP may reduce the number of infants considered to need ROP screening. These models do not offer 100% sensitivity in detecting persistent stage 3 ROP into zone III of the retina [5]. Nor do they enable an infant at risk of severe ROP to be excluded from screening. Some authors argue that predictive models should be clearly defined, reproducible and not open to subjective interpretation, as may be the case with image-based assessment [5].

In our study, after the first ophthalmoscopic examination and follow-up with BIO, ROP staging is determined and the need for treatment is assessed. The study variables of GA, BW and postnatal weight gain are then excluded from the model, to be replaced by those presenting the greatest significance, i.e., the retinal avascular area [5] and the rate of postnatal vascularisation, in the view that these variables play a primary role in the pathophysiology of ROP.

The GA and BW variables included in the ROP predictive models and obtained before the first fundus examination are time-related. Information obtained at a later stage includes the avascular area of the retina [36], the duration of mechanical ventilation, the rate of postnatal weight gain, the number of transfusions required and the presence or otherwise of sepsis. Finally, in the following weeks, a note is taken of the ROP stage observed, preplus disease, plus disease and the rate of vascularisation. Preplus disease is defined as retinal vascular dilation and tortuosity that is abnormal, but insufficient for plus disease.

All predictive models of treated ROP are subject to considerations of time elapsed. In our study population, about 50% of children with GA ≤ 24 weeks were treated [6]. Our predictive model obtained following the first examination includes the avascular area (DD) and the duration of mechanical intubation [6]. Finally, the developmental model is ascertained following an additional 3–4 weeks postnatal, in order to determine the rate of vascularisation (DD/w).

The model we present unifies the consideration of all patients requiring treatment for ROP. In aggressive ROP, Padhi observed a slow rate of vascularisation [7]. Patients treated with anti-VEGF usually present a large avascular area and delayed vascularisation [37]. Those with type 1 ROP fit this pattern, while patients presenting persistent type 2 ROP that requires treatment usually have a very slow rate of vascularisation. In the ETROP study, these eyes that had a risk of ≥15% were termed “high-risk” prethreshold [26]. In risk Table 3 of our model, some eyes have a temporal avascular area of 2–3 DD and a risk > 15%. This may explain why in some cases we try persistent stage 3 ROP. Our model does not assess aberrant neovascularisation.

The avascular area is greater in type 1 ROP than in type 2. The administration of 0.25 mg of Bevacizumab may produce a sufficient increase in the rate of vascularisation and lead to the regression of ROP. On the other hand, it may be insufficient and provoke reactivation of the ROP [37]. An insufficient rate of vascularisation (<0.5 DD/week) implies an ROP treatment risk that is 19 times greater than that of patients with a vascularisation rate of ≥0.5 DD/w. This consideration is illustrated in Table 2 and Table 3.

In the model we present, some of the risk factors considered may be modifiable. For example, reducing the duration of mechanical ventilation affects the model both directly and indirectly by increasing the rate of vascularisation. Moreover, greater postnatal weight gain will produce a faster vascularisation of the retina [38]. Other risk factors, such as the need for blood transfusions or the presence of sepsis, are significant in the univariate analysis but disappear in the multivariate analysis. However, the avascular area (space) and the rate of vascularisation are physical values that are difficult to modify, at least until we obtain a better understanding of the association between cytokines or other biomarkers and that of modifiable risk factors with these two variables.

A paediatric ophthalmologist may administer 2000 laser impacts within the periphery of the retina when the pupil measures 5–6 mm. In this context, indenting the periphery and evaluating the avascular area is relatively straightforward. If in two weeks the vascularisation has not advanced by 1 DD and in four weeks by about 2 DD (<0.5 DD/w), and if the initial avascular area is >4 DD, the ischaemia might not be compensated sufficiently to prevent fibrovascular proliferation. In extensive avascular areas, not even a good rate of vascularisation (≥0.5 DD) would be sufficient to inhibit the proliferative process. This situation is observed in treatment with anti-VEGF in zone I, when twins experience foetal distress and when there is anoxic pathology of the umbilical cord, with mature children of GA > 30 weeks who have had little or no mechanical ventilation.

The limitations of the study include its retrospective design. The algorithm needs to be validated in other study populations. Nonexpert paediatric ophthalmology needs a learning curve of the scleral indentation technique. This increases the time needed for ophthalmologists and the risk of bradycardia. Among the strengths, throughout the study period, we always performed the same exploration and indentation technique with BIO. It helps to better explore the “posterior zone II” that extends 2 DD into zone I. We include new physical variables linked to the predictive model: the avascular area and the weekly vascularization rate of the temporal retina. They can help generate a more universal model not so linked to gestational age and birth weight.

Among the premature infants who meet the inclusion criteria we describe, our model seeks to identify those who may require ROP treatment. For this reason, we establish a low level of sensitivity (60%) and a high level of specificity (95%). This model is intended to assist paediatric ophthalmologists at the early stage of ROP treatment. Alternatively, our model could be used to complement others, with high sensitivity (93–100%) and low specificity (28–46%), or to help provide a basis for deciding to treat or continue observing persistent avascular stage 3 ROP into zone III of the retina.

## 5. Conclusions

We present a model to predict the need for ROP treatment. This model has only three variables: avascular area (DD) in the first examination, intubation time and rate of vascularisation. The model presents two cut-off points at which the risk increases significantly: an avascular area >4 DD and a rate of vascularisation on the temporal retina <0.5 DD/w. Although the model is simple to apply, it may be affected by bias in subjective image registration or in evaluation with the BIO. However, a trained paediatric ophthalmologist capable of applying laser treatment to a premature infant can readily assess the avascular area and the reduction achieved. This examination is usually conducted at two-week intervals. Hence, the rate of vascularisation can easily be determined.

## Figures and Tables

**Table 1:** Multivariate analysis of risk factors for developing ROP requiring treatment.

Risk Factors	*p* Value	Odds Ratio (95% CI)
Duration of invasive respiratory support, days	0.001	1.05 (1.02–1.08)
Each additional diameter of avascular area (DD)	0.000	2.21 (1.66–2.94)
Retinal vascularisation rate, <0.5 vs. ≥0.5 DD/week	0.000	19.03 (6.52–55.55)

Abbreviations: ROP: retinopathy of prematurity; DD: diameter of the disc.; w: week.

**Table 2:** Risk of ROP requiring treatment for infants with vascularisation rate ≥0.5 DD/week [26,27]. The variables used are: temporal avascular area vs. duration of invasive respiratory support.

Duration of Invasion Respiratory Support (Days)	Avascular Area of the Retina in the First Examination (DD)									
	1	2	3	4	5	6	7	8	9	10
0	0.17%	0.38%	0.84%	1.85%	4.00%	8.44%	16.95%	31.10%	49.95%	68.84%
5	0.22%	0.49%	1.09%	2.39%	5.15%	10.73%	21.01%	37.05%	56.56%	74.23%
10	0.29%	0.65%	1.42%	3.10%	6.61%	13.52%	25.75%	43.41%	62.92%	78.96%
15	0.38%	0.84%	1.85%	4.00%	8.45%	16.96%	31.13%	50.00%	68.86%	83.03%
20	0.50%	1.09%	2.40%	5.16%	10.74%	21.03%	37.07%	56.58%	74.24%	86.44%
25	0.65%	1.42%	3.10%	6.62%	13.56%	25.76%	43.43%	62.94%	78.98%	89.26%
30	0.84%	1.85%	4.01%	8.46%	16.98%	31.15%	50.02%	68.89%	83.04%	91.55%

The risk of requiring treatment is represented by the colour of the cells, with green cells indicating a risk of less than 10%, yellow indicating a risk of 10–15%, and red cells indicating a risk of 15% or higher. Abbreviations: DD, diameter of the disc.

**Table 3:** Risk of ROP requiring treatment for infants with vascularisation rate <0.5 DD/week. The variables used are: temporal avascular area vs. duration of invasive respiratory support.

Duration of Invasion Respiratory Support (Days)	Avascular Area of the Retina in the First Examination (DD)									
	1	2	3	4	5	6	7	8	9	10
0	3.20%	6.83%	13.95%	26.40%	44.25%	63.71%	79.52%	89.57%	95.00%	97.67%
5	4.14%	8.72%	17.45%	31.86%	50.84%	69.59%	83.50%	91.80%	96.12%	98.20%
10	5.33%	11.07%	21.60%	37.87%	57.41%	74.89%	86.84%	93.58%	96.99%	98.61%
15	6.83%	13.96%	26.42%	44.27%	63.73%	79.54%	89.58%	95.00%	97.67%	98.93%
20	8.73%	17.46%	31.88%	50.87%	69.61%	83.52%	91.81%	96.12%	98.21%	99.18%
25	11.08%	21.61%	37.89%	57.44%	74.91%	86.85%	93.59%	96.99%	98.62%	99.37%
30	13.97%	26.44%	44.29%	63.76%	79.55%	89.59%	95.01%	97.68%	98.93%	99.51%

The risk of requiring treatment is represented by the colour of the cells, with green cells indicating a risk of less than 10%, yellow indicating a risk of 10–15%, and red cells indicating a risk of 15% or higher. Abbreviations: DD, diameter of the disc.

## Data Availability

The authors declare that the data in this research are available from corresponding authors upon reasonable request.
